# Dynamics of phagocytosis mediated by phosphatidylserine

**DOI:** 10.1042/BST20211254

**Published:** 2022-10-25

**Authors:** Daan Vorselen

**Affiliations:** 1 Department of Biology, University of Washington, Seattle, WA 98105, U.S.A.; 2 Department of Cell Biology and Immunology, Wageningen University, Wageningen 6708PD, the Netherlands

**Keywords:** cytoskeleton, efferocytosis, immune receptors, mechanobiology, phagocytosis, phosphatidylserine

## Abstract

Phagocytosis triggered by the phospholipid phosphatidylserine (PS) is key for the removal of apoptotic cells in development, tissue homeostasis and infection. Modulation of PS-mediated phagocytosis is an attractive target for therapeutic intervention in the context of atherosclerosis, neurodegenerative disease, and cancer. Whereas the mechanisms of target recognition, lipid and protein signalling, and cytoskeletal remodelling in opsonin-driven modes of phagocytosis are increasingly well understood, PS-mediated phagocytosis has remained more elusive. This is partially due to the involvement of a multitude of receptors with at least some redundancy in functioning, which complicates dissecting their contributions and results in complex downstream signalling networks. This review focusses on the receptors involved in PS-recognition, the signalling cascades that connect receptors to cytoskeletal remodelling required for phagocytosis, and recent progress in our understanding of how phagocytic cup formation is coordinated during PS-mediated phagocytosis.

## Introduction

In a classical paper by Fadok et al. exposure of phosphatidylserine (PS) was identified as a molecular trigger for clearance of apoptotic lymphocytes by macrophages [1]. Phosphatidylserine (PS) is now firmly established as a key trigger for phagocytosis, defined as the uptake of large (⌀ > 1 µm) particles by cells. PS is arguably still most well-known for its specific role in uptake of apoptotic cells, a process also termed efferocytosis. This process is common to many cell types, and is carried out by professional phagocytes (e.g. macrophages and dendritic cells) and regular tissue cells (e.g. epithelial and endothelial cells) alike [2]. Besides efferocytosis, PS has many additional functions. This includes intercellular signalling in a broad range of cell–cell interactions, for instance phagocytosis of non-apoptotic dying cells [3, 4], partial uptake of live cells (such as in synaptic pruning [5]), fertilization [6], cell-to-cell clustering by T cells [7], and even cell–cell fusion [8]. PS has further been reported to change the biophysical properties of membranes [9] and to regulate intracellular signalling and trafficking [10]. These intracellular functions predominantly take place in healthy cells, where PS is generally predominantly present in the internal leaflet of the cell membrane.

During apoptosis PS becomes increasingly exposed on the external membrane leaflet, due to changed activity of scramblases and flippases [11]. Interestingly, exposure of PS triggers primarily a tolerogenic form of uptake which, in addition to clearance and degradation of the target, is associated with anti-inflammatory signalling [12]. This combination of triggering phagocytosis and anti-inflammatory signalling is abundantly exploited by viruses, bacteria and protozoan parasites, which expose PS as a form of apoptotic cell mimicry [12]. The proper clearance of dying and dead cells itself also has great physiological importance during development, regular tissue maintenance, and infection [13–15]. Failure in efferocytosis is associated with multiple disease conditions, including auto-immune disorders, neurodegenerative disease, and atherosclerosis [16]. Modulating efferocytosis has also become an attractive target for therapeutic intervention [17]. Increasing targeted efferocytosis, for example, yields promising results in mouse models of atherosclerosis [18]. Inhibiting efferocytosis may, on the other hand, be desirable for creating a more inflammatory environment that promotes anti-tumour responses [12, 19].

Impressive progress has been made over the past two decades in identifying the receptors mediating PS recognition during phagocytosis. With a multitude of identified PS-receptors, the question now becomes which receptors function in which physiological contexts and how receptors inputs are integrated to elicit downstream cellular responses. Moreover, how the activity of the cytoskeleton, which drives the formation of the phagosome, is coordinated in space and time in PS-mediated phagocytosis is poorly understood compared with phagocytosis driven by opsonins (antibodies or complement) [20]. This is particularly intriguing given the broad roles of PS in fundamentally different cell–cell interactions such as synaptic pruning [5], clustering of T cells [7], and cell–cell fusion [8]. How, at least partially, similar molecular signals and recognition receptors result in such distinct cellular behaviours is poorly understood. This review focusses on the known receptors involved in PS recognition, the signalling cascades that lead to cytoskeletal remodelling downstream of these receptors, and the orchestration of cytoskeletal reorganization during PS-mediated phagocytosis.

## Recognition of phosphatidylserine

**Figure 1.**
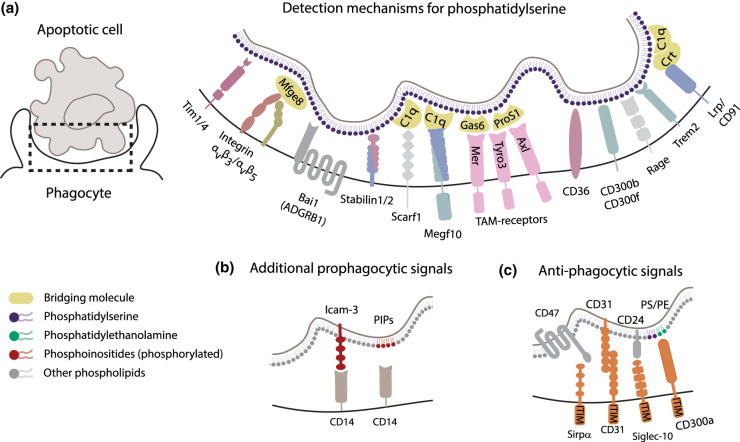
Engulfment and don't eat me signals during phosphatidylserine-mediated efferocytosis. (**a**) Receptors involved in PS recognition. (**b**) PS-independent recognition of apoptotic cells. (**c**) Anti-phagocytic (‘don't eat me’) signalling. Receptor colours correspond to their known involvement in downstream signalling pathways, with those visualized in two colours present in multiple pathways (see Figure 2).

More than 15 distinct cell surface proteins that directly or indirectly - through bridging molecules - mediate binding to PS have now been established in mammals (Figure 1a). The evidence supporting the involvement of these receptors varies between biochemical assays using purified proteins, genetic deletion, ectopic expression models or PS liposome competition assays. Ideally, a combination of such approaches is used to show both PS recognition and a role in PS-dependent phagocytosis. Among the earliest identified direct receptors for PS was brain-specific angiogenesis inhibitor BAI-1 [21]. Although BAI1 has been convincingly shown to both mediate PS recognition and induce downstream signalling, its general importance in macrophage biology was recently contested because of its low expression levels in macrophages [22]. Multiple T-cell immunoglobulin and mucin domain-containing proteins, most notably Tim1, which is also known as Kim1 [23], and Tim4, also directly bind PS and are involved in PS-mediated phagocytosis [24]. Many other phagocytic receptors for PS, including Stab1 & 2 [25], Rage [26], Scarf1 [27]**,** CD36 [28], CD300 family proteins CD300b [29] and CD300f [30] and Trem2 [31] are scavenger receptors or generally quite promiscuous. These receptors may bind a wide variety of ligands in addition to PS, often including other negatively charged lipids, apolipoproteins, bacteria, and more [32, 33].

In addition to receptors that directly bind PS, multiple bridging molecules enable indirect interactions between phagocytes and PS-exposing targets (Figure 1a). Mfge8 can bridge PS on the target surface to the RGD motif on phagocytic integrins, particularly integrin α_v_β_3_ and α_v_β_5_ [34]. Similarly, Gas 6 can bind PS [35], and engage macrophages through their Mer(TK) receptor [36]. Gas6 can also bind to Tyro3 and Axl, which together with Mer belong to a family named TAM-receptors [37]. Protein S, which has homology to Gas 6, similarly bridges PS to Mer and Tyro3 [38, 39]. Yet additional bridges can be formed by complement component 1q (C1q) between PS and Megf10 [40, 41], of which the homologues Ced-1 in *Caenorhabditis elegans* and Draper in *Drosophila melanogaster* have extensively been studied [42, 43]. Finally, calreticulin (Crt) is a bridging molecule that can bind PS directly, or through association with C1q and is recognized by Lrp1 (CD91) [44, 45]. In addition to a role in uptake of apoptotic cells, calreticulin plays an important role during phagocytosis of live cancer cells [46].

It is noteworthy that many PS receptors are regulated by sheddases, enzymes with the ability to cleave off the ectodomain of membrane proteins. Tim1/4, Trem2, Mer, CD36 and Lrp1 can all be shed from the cell surface, most notably by Adam 17 activity [47–51]. Shedding of PS receptors has a dual inhibitory effect on PS recognition and efferocytosis. Firstly, the obvious direct effect that the receptor is removed from the phagocyte's surface. Secondly, once in soluble form the ectodomains may mask PS exposed on apoptotic cells or by sequestering bridging molecules. The presence of multiple PS receptor ectodomains in elevated levels is associated with disease, including neurodegeneration and auto-immune diseases [52, 53]. Soluble PS receptors may also have additional physiological functions, such as inducing inflammatory responses and increasing phagocyte survival, such as in the case of Trem2 [54].

Physiological preys that expose PS often display additional phagocytosis-promoting molecules (Figure 1b). CD14, a GPI-anchored protein that lacks an intracellular signalling domain, is involved in uptake of apoptotic cells [55], which it may recognize through Icam-3 or phosphorylated phosphoinositides (PIPs) [56, 57]. Like PS, PIPs also lose their asymmetric distribution and become exposed on the external membrane leaflet during apoptosis. Although PS is far more abundant, making up to 10% of the plasma membrane phospholipids [58], increased PIP exposure was recently reported to contribute to triggering efferocytosis [56]. To further add to the complexity, in addition to prophagocytic ligands, anti-phagocytic ligands, or ‘don't eat me’ signals also play a key role in meal selection by macrophages (Figure 1c). PS exposure alone is often insufficient to trigger uptake of cells, and anti-phagocytic ligands need to be removed from the target cell or blocked before efficient uptake takes place. Receptors for antiphagocytic ligands often contain an immunoreceptor tyrosine-based inhibitory motif (ITIM) domain and were recently reviewed in detail [59]. These ‘don't eat me signals’ include CD47 and CD24, which are recognized by SIRPα and Siglec-10 [60], respectively, and CD31, which engages in homotypic interactions. The CD300 protein family, which includes multiple prophagocytic receptors, also includes inhibitory receptors, specifically CD300a, which, surprisingly, has been suggested to, amongst others, recognize PS [61].

## Signalling pathways eliciting downstream cytoskeletal rearrangements

**Figure 2.**
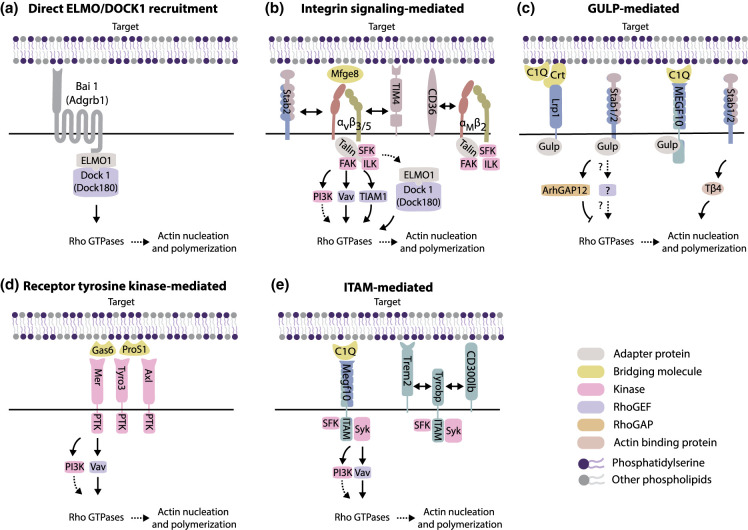
Signalling pathways leading from PS receptor engagement to actin reorganization. (**a**–**e**) Pathways linking PS receptors to actin reorganization, where individual panels indicate distinct initial signalling steps. Receptors visualized in two colours are present in multiple pathways (and hence multiple panels). Solid lines indicate more direct connections than dashed lines.

Engagement of PS receptors during efferocytosis triggers two main processes: immune tolerance and phagocytosis. The former includes the release of anti-inflammatory cytokines (including interleukin (IL) 10 and TGFβ) and inhibition of release of pro-inflammatory cytokines (e.g. TNFα, IL-1β and IL-12), and has been reviewed in detail before [12, 37, 62, 63]. Instead, we will focus here solely on how PS recognition results in the signalling events that lead to downstream cytoskeletal remodelling and ultimately target engulfment. In these signalling pathways, Rho-GTPases, like Rac1, Rac2, RhoA, and RhoG, play a central role [64]. As master regulators of cytoskeletal remodelling, they coordinate the large-scale actin rearrangements required for phagocytic cup formation. Indeed, multiple signalling pathways link PS receptors to Rho-GTPase activity (Figure 2).

A well-established pathway connects direct PS-receptor Bai1 to Rac1 activation (Figure 2a). Bai1 directly binds the adaptor protein Elmo1, which forms a complex with Dock1 (also known as Dock180), a guanine nucleotide exchange factor (GEF) that can activate Rac1 [21]. This pathway was initially established, and shares great similarity with, engulfment pathways in *C. elegans* [65]. Other receptors, including integrin α_v_β_5_ and Stabilin (Stab) 2, through its association with integrin α_v_β_5_, also make use of this Elmo1-Dock1 signalling module [66, 67] (Figure 2b). A second classical pathway leads to Rho GTPase activation through Gulp (Ced-6 in *C. elegans*) and was also originally identified in *C. elegans*. [65, 68, 69]. Gulp has been implicated downstream of indirect PS receptors Megf10 and Lrp and direct PS receptor Stab2 [69–71], yet how Gulp regulates Rac1 activation remains to be clarified (Figure 2c). Recently, Gulp was established to directly interact with Rac1 inactivating protein ArhGAP12 [72]. Interestingly, Stab2 also directly interacts with actin sequestering protein thymosin β4, and may therefore also have an additional, rather direct, impact on cytoskeletal organization [73].

Because of the large number of PS-recognition receptors, the involvement of additional downstream signalling pathways is likely. Indeed, integrins involved in PS phagocytosis for example, may use pathways independent of Elmo1-Dock1 to activate Rho GTPases (Figure 2b). This involves classical integrin signalling molecules such as focal adhesion kinase (FAK) and integrin-linked kinase (ILK), which can lead to downstream activation of multiple RhoGEFs [74]. For example, in endothelial-like trabecular meshwork cells, after engagement of integrin α_v_β_5_ the GEF Tiam1, and not Dock1, appeared critical for phagocytosis [75]. Similarly, in macrophages, the GEF Vav3 has been reported as a downstream Rho GTPase activator of integrin α_v_β_5_ and direct PS-receptor TIM4. The VAV family of GEFs, in particular Vav1, has also been shown to be involved after indirect PS engagement by the TAM receptors (Tyro3, Axl, Mer) (Figure 2d). These receptors belong to the family of receptor tyrosine kinases that have an intracellular catalytic domain directly engaged in kinase activity. Mer can bind Vav1 directly and release it upon phosphorylation [76].

Possibly underappreciated in the context of efferocytosis is that multiple PS-receptors may also use pathways with similarity to antibody (Ab)-mediated phagocytosis (Figure 2e). In Ab phagocytosis, Fc-receptors are engaged on the cell surface. These receptors contain an intracellular ITAM domain, which can be phosphorylated by Src family kinases (e.g. Lyn), after which they induce a signalling cascade through Syk [20]. In turn, this leads to the activation of RhoGEFs, as well as PI3 Kinase and Plcγ [20]. Multiple direct or indirect receptors of PS either contain an ITAM (such as Megf10) [77] or associate with the ITAM containing coreceptor Tyrobp (Dap12), such as Trem2 and CD300lb [29, 78]. The potential importance of this pathway is underscored by two recent studies. First, using a genome-wide screening approach for identification of regulators of PS-mediated phagocytosis in a murine macrophage-line, the receptor Trem2 and downstream effectors including Lyn, Syk, and PI3 kinase were found to be critical in PS-mediated phagocytosis [79]. Second, in work by Morrissey et al. chimeric antigen receptors for phagocytosis (CARPs) were developed in macrophages. Only those CARPs containing ITAM domains, and not those based on the internal signalling moieties Bai1 or Mer, were able to efficiently trigger phagocytosis [80].

## Receptor cooperation: tether and tickle?

Why are there so many different receptors for PS with apparently redundant functionality? Multiple hypotheses to explain redundancy have been put forward. Firstly, it is likely that receptor expression and hence involvement in phagocytosis is, at least partially, cell type specific. Indeed, multiple studies reported cell-specific involvement of receptors, such as Trem2, which has been specifically associated with clearance of neuronal debris by microglia, the tissue-resident macrophages of the brain [81, 82]. Differences in involvement or expression of receptors have also been reported between macrophages and dendritic cells [83], or between astrocytes and microglia [40]. Nonetheless, single phagocytes appear to use multiple receptors, such as microglia which, in addition to Trem2, also require Bai1 and Tim4 for efficient phagocytosis [84].

The requirement of multiple receptors could also indicate distinct functioning of individual receptor species during phagocytic cup formation. A central hypothesis in PS-mediated phagocytosis is that some receptors play a role merely in establishing adhesion between phagocyte and target cell (‘tethering’), whereas other receptors lead to tethering as well as downstream signalling (‘tickling’) [85]. This hypothesis is attractive because of its simplicity and was initially inspired by the observation that the intracellular domain of PS-receptor Tim4 is dispensable for efferocytosis. It was therefore suggested that Tim4 only mediates tethering and does not mediate signalling [86, 87]. Tethering and tickling were further suggested to be two distinct consecutive steps during apoptotic cell phagocytosis [88]. From recent studies of antibody (Ab)-mediated phagocytosis it has, however, become clear that even during the first steps of contact formation signalling is essential. Such early signalling events enable changes in the cortical actin cytoskeleton and thereby increased mobility, engagement and clustering of receptors [89]. Furthermore, it is difficult to exclude that supposed tethering receptors truly have no signalling function. Receptors with dispensable, or even no, intracellular domain may still associate, through their extracellular or membrane domain, with other membrane proteins that mediate intracellular signalling (coreceptors). Indeed, multiple phagocytic receptors that recognize PS, including Trem2 and CD300b, associate through their transmembrane domain with Tyrobp, a protein containing an intracellular immunoreceptor tyrosine-based activation motif (ITAM), which mediates intracellular signalling [29, 31]. Direct and indirect association with integrins as coreceptors has even been reported for Tim4 [90, 91], the receptor that sparked the tethering hypothesis. An *in vivo* imaging approach further revealed that Tim4 deficient microglia still formed phagosomes, albeit of reduced stability, meaning that the pseudopods surrounding the target extended and retracted repeatedly [84]. Such observations are inconsistent with a pure tethering role for Tim4.

Although distinct functioning of receptors is extremely likely, considering these recent findings the classical tethering and tickling model likely needs to be reassessed. Recent advances in imaging approaches now allow tracking the localization and dynamics of receptors and key cytoskeletal proteins over the course of phagocytosis [92], and even allow following the mechanical progression of phagocytosis [93, 94]. The more detailed readout of phagocytosis that such approaches provide, could be key in dissecting the individual, and cooperative, contributions of PS-receptors to signalling and phagocytic cup shaping mechanisms.

## Beyond Rac1 activation: dynamics of phosphatidylserine-mediated target internalization

Engulfment of targets in phagocytosis is ultimately driven by remodelling of the actin cytoskeleton [89, 95, 96]. Building a phagocytic cup requires dynamic and large-scale assembly, as well as disassembly of actin filaments. These processes need to be carefully coordinated in space and time for successful engulfment, and are regulated by a multitude of actin-binding proteins. The exact regulation and timing of the activity of these regulators can dramatically affect the dynamics of uptake, and distinct uptake modes have been described for different targets.

In Ab-mediated phagocytosis, for which the engulfment process has been studied in most detail, F-actin-filled protrusions are important for initiating target engagement. Early signalling events lead to a disruption of the actin cortex of the cell, which allows increased mobility and thereby clustering of receptors [89]. Thin membrane protrusions then grow outward from the cell cortex and tightly surround the target. The physical forces required for shaping phagocytic cups are generated by Arp2/3-dependent actin polymerization and Myosin-II activity. These forces are transduced to the target, resulting in local target constriction at the rim of the phagocytic cup [94, 97, 98]. Protrusion and constriction at the cup rim ultimately bring together the pseudopods at the opposite end of the target, where membrane fusion machinery closes the phagosome [99]. During engulfment, actin is depolymerized at the base of the phagocytic cup to allow passage of the target through the cortical actin layer of the cell, as well as enabling fusion of vesicles for delivery of the required membrane for expansion of the phagocytic cup [20]. The coordination of the activity of various actin-binding proteins, leading to local and timely assembly and disassembly of actin filaments is regulated by multiple guanine nucleotide exchange factors (GEFs) and activating proteins (GAPs) of Rho GTPases [64], and lipid signalling by phosphoinositides [100]. Different mechanisms of phagocytic cup formation, in which outward protrusions are largely lacking, and targets appear deeply embedded in the cytoplasm before cup closure, leading to the name ‘sinking’ phagocytosis, have also been described [101]. This mode of uptake has classically been associated with complement-mediated phagocytosis, although this view has recently been contended [102, 103].

Little is known regarding the dynamics of PS-mediated engulfment, and it is currently unclear if it resembles one of these previously described strategies, or perhaps, progresses yet in different fashion. Furthermore, it is largely unknown which actin-binding proteins are involved, and how their activity is coordinated in space and time by upstream regulators. Recent microscopy studies, however, give some indications of cup shaping mechanisms during PS-mediated phagocytosis. Uptake of PS-coated beads by a murine macrophage line resembled ‘sinking’ phagocytosis described above, and further reported involvement of long finger-like F-actin protrusions in early stages of engulfment [79]. Studies in *Drosophila melanogaster* indicate that the uptake strategy used, and specifically the use of long pseudopods, may depend on the physical surroundings where phagocytosis takes place [104, 105]. PS-mediated uptake of apoptotic cells by epithelial cells in early zebrafish embryo's revealed outgrowing cups with F-actin accumulation and target constriction at the rim, reminiscent of Ab-mediated uptake as observed by macrophages *in vitro* [106]. This study also revealed that, *in vivo*, phagocytosis of apoptotic cells can involve piecemeal uptake, or trogocytosis [107], in which parts of apoptotic cells are ‘nibbled’ off and engulfed. These studies revealing apparently distinct engulfment mechanisms indicate that the engulfment dynamics in PS-mediated phagocytosis may depend on the cell type, the environmental context, and additional cues presented by the target.

## New physically accurate model targets for studying efferocytosis

Phagocytosis is an intricate process that requires rapid recognition, lipid and protein signalling, and cytoskeletal remodelling. Efferocytosis, in particular, is astoundingly complex due to the involvement of many ligands, a multitude of receptors for individual such ligands, as well as the involvement of anti-engulfment signals. Moreover, in *in vivo* studies, indirect effects on phagocytosis, such as effects on phagocyte proliferation and survival, as for example reported in the case of Trem2 in microglia [108], can be hard to discern from direct involvement of receptors in phagocytosis. To reduce this complexity, model targets that capture key attributes of apoptotic cells have frequently been used. Although necessarily a simplification, such systems may be essential to tease apart the signalling networks involved in PS phagocytosis.

As an early model system to study efferocytosis, bare carboxylate beads were used to mimic apoptotic cells [21]. Such beads carry a negative charge, but lack any resemblance of the molecular identity of prophagocytic ligands like PS. It is now quite achievable to coat glass or polystyrene beads with phospholipid bilayers, and the composition of such bilayers can be tuned to incorporate a physiological amount of PS [109]. We, and others, have recently established deformable microparticles that can be functionalized with a variety of ligands [93, 110]. A key benefit of such particles is that they can be made to accurately mimic physical properties, like size and rigidity, of apoptotic cells, whereas glass or polystyrene beads are 1–10 million times more rigid than apoptotic cells. Such new model systems will therefore also be key to achieving a better understanding of how phagocytes deal with the specific physical challenges in phagocytosis of apoptotic cells.

## Physical challenges in PS-mediated phagocytosis

PS-exposing targets, such as apoptotic cells, present unique physical challenges for the phagocytes engulfing them. Apoptotic cells are large, but also relatively soft, with an apparent Young's modulus (a measure for the resistance of a material to withstand uniaxial compression or elongation) of 0.1–10 kPa [95]. Many natural *(*e.g. bacteria) and lab-based model targets are thousand to more than million-fold more rigid [95]. This is especially relevant because phagocytosis is a mechanosensitive process, reportedly being more efficient for rigid than softer targets [93, 102, 111, 112]. Rigidity-dependent uptake has, to my knowledge, not been shown for PS-mediated phagocytosis specifically. However, the list of immune receptors that are identified as mechanosensitive molecules is rapidly expanding [113]. Particularly the involvement of integrins (Figure 2b), which are known to be broadly involved in mechanosensing in cellular processes [102, 114], in PS-mediated phagocytosis makes target rigidity dependence likely. Potential differences in degree, or range, of mechanosensitivity of specific PS receptors may even lead to differential engagement of distinct PS receptors of physically distinct targets. Future studies will need to confirm such differences and may thereby contribute to our understanding of target-specific signalling responses and the apparent redundancy among PS receptors.

It is also expected that cells undergo mechanical changes during apoptosis, for example because of disassembly of the F-actin cortex in late-stage apoptosis [115]. A decrease in rigidity has indeed been observed in single cells during apoptosis [116]. Surprisingly, this would likely present a mechanical anti-phagocytic signal [95]. It may, however, also allow phagocytes to protrude deep into apoptotic cells and thereby mediate partial engulfment, as recently observed for soft artificial microspheres [79]. An interesting feature of apoptotic cells is that they, for example due to the occurrence of blebs, are mechanically heterogeneous and also have a complex geometry [115]. Similar to target rigidity, phagocytosis can be affected by the geometry of the target, including size, shape and local curvature [95]. Observation of phagocytosis of geometrically complex targets suggests that phagocytes use local changes in curvature to adapt phagocytic strategies during engulfment, for example switching from whole target phagocytosis to partial engulfment (trogocytosis) [117]. How variation in mechanical properties of a single target may affect phagocytosis is currently unknown, but may include similar adaptations of engulfment mechanisms. To elucidate the strategies for dealing with mechanically heterogeneous and geometrically complex apoptotic cells, novel sophisticated experimental approaches are required and future studies should focus on capturing the dynamics of target engulfment in progress.

## Perspectives

- PS-mediated clearance of dying and dead cells has great physiological importance during development and regular tissue maintenance. Modulation of this process is a promising therapeutic strategy in the context of atherosclerosis, neurodegenerative disease and cancer.
- The individual and joined contribution of PS receptors to phagocytosis are likely more complex than suggested in the classical ‘tethering and tickling’ model. Multiple signalling pathways, including one closely resembling pathways of antibody-mediated phagocytosis, connect receptor engagement to Rho GTPase activation and cytoskeletal remodelling.
- Novel imaging and biophysical approaches allow observation of how phagocytic cups are built in space and time and simultaneously pinpoint where key molecules localize. These approaches may be key to deciphering how PS receptors work individually and in concert.

## Abbreviations

CARPs: chimeric antigen receptors for phagocytosis
GEF: guanine nucleotide exchange factor
IL: interleukin
ITAM: immunoreceptor tyrosine-based activation motif
TAM: Tyro3, Axl, Mer
